# Sex-Based Differences in Transcranial Doppler Ultrasound and Self-Reported Symptoms After Mild Traumatic Brain Injury

**DOI:** 10.3389/fneur.2019.00590

**Published:** 2019-06-11

**Authors:** Corey M. Thibeault, Samuel Thorpe, Nicolas Canac, Seth J. Wilk, Robert B. Hamilton

**Affiliations:** Neural Analytics, Inc., Los Angeles, CA, United States

**Keywords:** sex differences, traumatic brain injury, vascular reactivity, CBF autoregulation, blood flow

## Abstract

The possibility of sex-related differences in mild traumatic brain injury (mTBI) severity and recovery remains a controversial subject. With some studies showing that female subjects suffer a longer period of symptom recovery, while others have failed to demonstrate differences. In this study, we explored the sex-related effects of mTBI on self-reported symptoms and transcranial Doppler ultrasound (TCD) measured features in an adolescent population. Fifty-eight subjects were assessed—at different points post-injury—after suffering an mTBI. Subjects answered a series of symptom questions before the velocity from the middle cerebral artery was measured. Subjects participated in breath-holding challenges to evaluate cerebrovascular reactivity. The Pulsatility Index (PI), the ratio of the first peaks (P2R), and the Breath-Hold Index (BHI), were computed. Linear mixed effects models were developed to explore the interactions between measured features, sex, and time since injury while accounting for within subject variation. Over the first 10 days post-injury, the female group had significant interactions between sex and time since injury that was not present in the TCD features. This is the first study to compare sex-related differences in self-reported symptoms and TCD measurements in adolescents suffering an mTBI. It illustrates the pitfalls clinicians face when relying on subjective measures alone during diagnosis and tracking of mTBI patients. In addition, it highlights the need for more focused research on sex-related differences in concussion pathophysiology.

## Introduction

The presence of sex-related differences in mild traumatic brain injuries or concussions (mTBI), is a contentious subject. Several studies have found increased symptoms in females (1–3), as well as increased length of recovery (4–6). In the case of Preiss-Farzanegan et al. (7), a difference existed in adults (18 years-old or older), but not in minors (17 years-old and younger). Conversely, others have failed to demonstrate increases in female symptoms at all (8–12). In general, the current state of the literature suggests that the existence of gender dependence in concussion recovery and severity is still an open question (13, 14).

One commonality between these studies however, is that they all rely on neurocognitive evaluations, patient symptoms, or physical performance testing. Although these have been shown to provide insight into concussion severity and prognosis, they do not objectively measure physiological changes resulting from concussive injury. Here, we present a study comparing features of the cerebral blood flow velocity (CBFV) as measured with Transcranial Doppler (TCD) ultrasound in addition to self-reported symptoms to investigate sex-related differences in concussion.

With recent research demonstrating abnormalities in cerebral blood flow after a mTBI, it is clear that the microvasculature is affected (15–24). In Thibeault et al. (23), the cerebral hemodynamic changes in adolescents between 14 and 19 years old after suffering a clinically diagnosed mTBI were assessed using TCD. In that study two distinct phases of hemodynamic alterations after a concussive injury were identified. In the initial phase, beginning within an hour of injury and lasting through the first 48 h, Pulsatility Index (PI), and peak ratio (P2R), showed a significant difference from controls. After 48 h however, these differences in pulsatile features were no longer observable. At this point in their recovery the breath-holding index (BHI), a measure of the cerebral vascular reactivity (CVR), was significantly increased when compared to controls. This lasted through day seven. After which, the population level increase was no longer significant.

Although Thibeault et al. (23) was the first study to suggest the presence of multiple phases of hemodynamic dysfunction, there have been others demonstrating measurable alterations in mTBI subjects using TCD. Utilizing a hypercapnia challenge, Len et al. (15) found significant changes in a population of concussed subjects. A subsequent study found significant differences during hypocapnia (24). Similarly, the study from Albalawi et al. (25), found vasoreactivity was linearly related to both severe headaches and cognitive symptoms. Baily et al. (18), found lowered CVR in a population of subjects suffering from chronic symptoms. The present study, however, appears to be the first to explore sex specific abnormalities in mTBI subjects with both self-reported symptoms and an objective physiological measure.

## Methods

### Patient Population

Participants in this study consisted of adolescents between 14 and 19 years old from the Los Angeles, California metropolitan area. Subjects classified with an mTBI were diagnosed by independent physicians and were scanned at different times post-injury. For this analysis these longitudinal measurements were restricted to 13 days post-jury from 58 unique subjects. The population was comprised of 37 male and 21 female participants, with 81 and 57 total exams for each group, respectively. Within the male group, 17 subjects had more than one scan during the course of recovery and a median number of scans of 1.0 with an IQR of 2.0. In the female group, 13 subjects had more than one scan and there as an overall median 2.0 scans with an IQR 3.0. The control group consisted of 109 age-matched subjects, 89 male and 12 female, who had no reported head-injuries in the preceding 12-months. The control group was only scanned a once. The study was approved by Western Institutional Review Board (IRB #20141111). This data was previously used in Thibeault et al. (23).

### Data Collection

**Figure 1:**
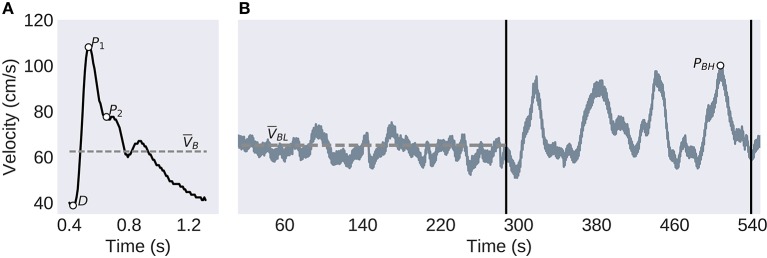
Experimental Protocol and TCD Analysis. **(A)** The individual pulses are extracted before the systolic peak (P1), diastolic trough (D), second peak (P2), and the mean velocity (V_*B*_) are identified. **(B)** The CVR protocol consists of a 5-min baseline, left of the first vertical bar, followed by the four breath-holding challenges, between the vertical bars. The low-frequency component of the global signal (solid gray line), is used to compute the baseline mean velocity (V_*BL*_, dashed line) and the largest peak velocity (P_*BH*_) used to calculate the BHI.

The TCD signals were acquired from the middle cerebral arteries (MCA) transtemporally by ultrasonographers utilizing 2 MHz probes held by an adjustable headset. End-tidal CO_2_ was collected concurrently through a nasal cannula. The exam protocol, illustrated in Figure 1, began with a 5-min baseline period of normal breathing. This was followed by a series of 4 breath-holding challenges as an estimate of CVR. Each of these consisted of a 25-s period where the subject was instructed to hold their breath, followed by 35-s of normal breathing.

### Symptom Reporting

Before each of the data collection session, subjects were asked to answer a number of questions similar to the graded symptom scale checklist. Table 1 presents the list of questions where subjects were asked to numerically rate their current symptom state. The ratings were used both individually and summed together as an estimate of severity.

**Table 1:** Subjects were asked score themselves on the following symptoms based on how they feel now–None (0), Mild (1,2), Moderate (3,4), Severe (5,6).

**Symptoms**
Headache
Pressure in head
Neck pain
Nausea or vomiting
Dizziness
Blurred vision
Balance problems
Sensitivity to light
Sensitivity to noise
Vision problems
Feeling like in a “fog”
“Don't feel right”
Difficulty concentrating
Difficulty remembering
Fatigue or low energy
Confusion
Drowsiness
Trouble falling asleep
More emotional
Irritability
Sadness
Nervous or anxious

### Analysis

The TCD features found to correlate with mTBI in Thibeault et al. (23), were used to compare with the self-reported symptoms. The first pulse level feature, extracted from the baseline section, was the PI. This is generally believed to be related to distal resistance however, it appears to be more modulated by a number of physiological processes (26), PI is found by

$$\begin{array}{l} PI=\;\left(P_{1}-D\right)\;/\;{\bar{\text{V}}}_{B}. \end{array}$$

Where *P*_1_, *D*, and V*_B_* are defined in Figure 1.

The second, P2R, is the ratio of P_2_, and P_1_, as illustrated in Figure 1. This has been hypothesized to be related to distal bed compliance (27). This is found by

$$\begin{array}{l} P2R\;=\;P_{2}/P_{1}. \end{array}$$

These features were individually averaged across all the extracted pulses from the baseline section.

The CVR was estimated using the BHI. This was found by first finding the highest peak of the low-pass filtered CBFV waveform between the four breath-hold sections as illustrated in Figure 1. This is then related to the baseline mean velocity by

$$\begin{array}{l} BHI\;=\frac{P_{BH}-\;V_{BL}}{V_{BL}}. \end{array}$$

### Statistical Modeling

Linear mixed-effect models were developed to explore the interactions between effects of time and sex on the measured variables while compensating for the unbalanced groupings and the potential individual subject variation. The models were developed in R using the lme4 package (28). Summary statistics and significance values—using the satterthwaite method of degrees of freedom and *t*-test—were computed with the lmertest package (29). Additional model analysis was completed with the Psycho library (30). Effects were considered significant if *p* < 0.05, and the reported beta was at least twice the standard error (SE).

The models were developed for each of the three TCD features as well as the summed symptom scores, as dependent variables. For symptoms and BHI, the random-effects were explored by fitting different models with the maximum likelihood method and comparing with the likelihood ratio test—the models with significant improvement were selected. The fixed-effects and interactions were similarly compared, and the final models were then fit with the restricted maximum likelihood method. The models for PI and P2R failed to converge with the maximum likelihood method, however, the restricted method did reach convergence. Because of this, the resulting models both used a similar structure, with days-post injury, sex, and their interactions as fixed effects, and subject specific intercepts as random effects. For the sex category, a contrast encoding of [0.5, −0.5] with males as the reference was employed. Similarly, a dummy encoding with the controls as the reference group was used for the days-post category. These were grouped similar to Thibeault et al. (23). Correlations between features were evaluated using the Pearson correlation coefficient and the sex dependent interactions of the resulting regression lines were explored using the ANCOVA method with a set of linear models fit with the ordinary least squares method from lme4 (28). A one-way ANOVA was conducted to compare the effect of sex and condition (case or control), on age using the StatsModels package (31) in Python.

## Results

### Population

There was no significant interaction between the effects of sex and condition on age [*F*_(1, 163)_ = 0.08, *p* = 0.78], or main effects of either sex [*F*_(1, 163)_ = 1.86, *p* = 0.17] or condition [*F*_(1, 163)_ = 0.004, *p* = 0.96]. All subjects identified as athletes, with the majority of males, 26 (74%) subjects from the mTBI group and 77 (87%) from the control group, playing football as their primary sport. The other subjects were split between rugby, soccer, basketball, baseball, lacrosse, ice hockey, and quidditch. Although within the female population soccer was the most popular, 10 (45%) from the mTBI group and 7 (75%) subjects from the control group, the overall spread was more diverse and included volleyball, dance, track, swimming, basketball, softball and cheer. Within the mTBI population there was a slight difference in the reported mechanism of injury. For the male population 33 (89%) subjects reported being injured playing a sport, while 4 (11%) did not provide a mechanism. Within the female population 16 (76%) identified their cause of injury from a sport, whereas 5 (24%) reported another mechanism or did not provide a cause.

### Symptoms

**Figure 2:**
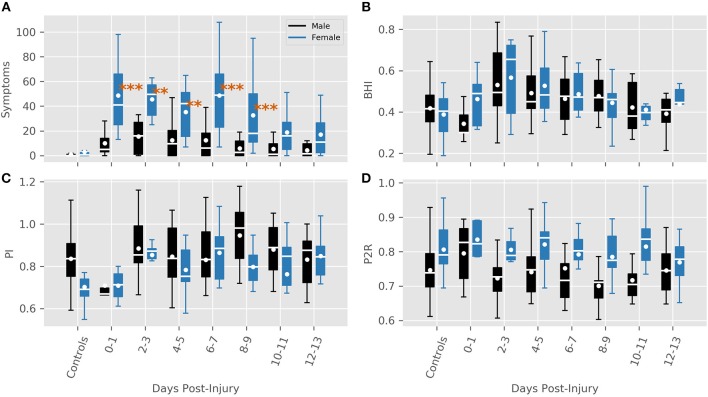
Features for each sex grouped by days post-injury. **(A)** Summed symptoms. **(B)** BHI. **(C)** PI **(D)** P2R. Significance values are indicated where a large interaction between sex and days-post was found (**P < 0.01, ***P < 0.001).

**Figure 3:**
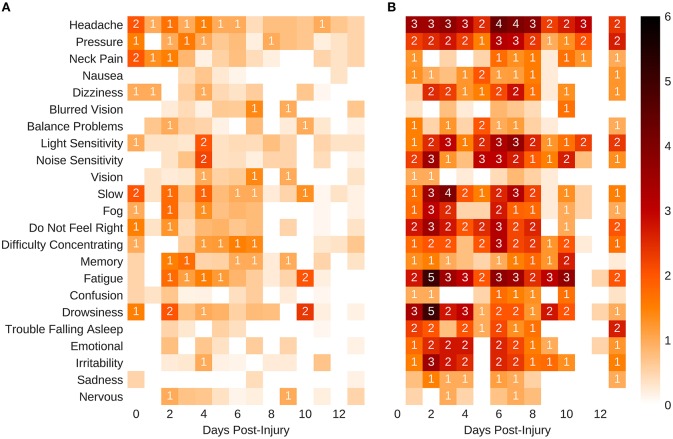
Self-reported mean symptom scores. **(A)** Males. **(B)** Females. The shaded squares without a value are below 1.

The model for symptoms had an explanatory power (conditional *R*^2^) of 76.74%, in which the fixed effects explain 49.95% of the variance (marginal *R*^2^). Though there was no overall main effect of sex in the model (β = 2.91, SE = 3.94, 95% CI [−4.58, 10.51], *t*_(215)_ = 0.74, *p* > 0.1), there were significant interactions between sex and days-post groups for the first 11 days, see Table 2. This is illustrated by the increased self-reported summed symptoms scores in the female population in Figure 2A. In addition, the individual symptom averages in Figure 3 illustrate that it was not a small subset of symptoms dominating the summed score for the female population. Additionally, there were large main effects for all days-post groupings, see Table 2.

**Table 2:** Mixed effect model results for symptoms.

**Variable**	**β (SE)**	**95% CI**	***t*(DF) *p***
(Intercept)	2.7 (1.97)	[−1.03, 6.52]	1.37(215) *p >* 0.1
Sex	2.91 (3.94)	[−4.58, 10.51]	0.74(215) *p >* 0.1
0–1 Days-Post	**27.54 (3.99)**	**[19.89, 35.06]**	**6.9(214)** ***p <*** **0.001**
2–3 Days-Post	**24.4 (3.5)**	**[17.65, 30.99]**	**6.97(222)** ***p <*** **0.001**
4–5 Days-Post	**18.84 (3.15)**	**[12.78, 24.78]**	**5.97(223)** ***p <*** **0.001**
6–7 Days-Post	**21.98 (3.01)**	**[16.01, 27.64]**	**7.29(230)** ***p <*** **0.001**
8–9 Days-Post	**13.34 (3.15)**	**[7.23, 19.28]**	**4.23(230)** ***p <*** **0.001**
10–11 Days-Post	**9.42 (3.25)**	**[3.21, 15.56]**	**2.9(228)** ***p <*** **0.01**
12–13 Days-Post	**8.56 (3.48)**	**[1.95, 15.14]**	**2.46(222)** ***p <*** **0.05**
Sex:0–1 Days-Post	**30.85 (7.98)**	**[15.43, 45.85]**	**3.87(214)** ***p <*** **0.001**
Sex:2–3 Days-Post	**20.4 (7.01)**	**[−1.08, 23.6]**	**2.91(222)** ***p <*** **0.01**
Sex:4–5 Days-Post	**16.88 (6.31)**	**[−2.29, 24.09]**	**2.67(223)** ***p <*** **0.01**
Sex:6–7 Days-Post	**27.98 (6.03)**	**[6.93, 33.6]**	**4.64(230)** ***p <*** **0.001**
Sex:8–9 Days-Post	**23.46 (6.31)**	**[4.82, 28.79]**	**3.72(230)** ***p <*** **0.001**
Sex:10–11 Days-Post	11.22 (6.49)	[−1.08, 23.6]	1.73(228) *p >* 0.05
Sex:12–13 Days-Post	10.92 (6.95)	[−2.29, 24.09]	1.57(222) *p >* 0.1

*The bold rows indicate significant effects or interactions*.

### TCD Features

#### BHI

The BHI model had a total a total explanatory power (conditional *R*^2^) of 45.16%, in which the fixed effects explain 13.83% of the variance (marginal *R*^2^). There was no main effect of sex found in the model, (β = −0.03, SE = 0.03, 95% CI [−0.09, 0.04], *t*_(215)_ = −0.73, *p* > 0.1), but there was one large interaction between sex and days-post at the 0–1 days grouping (β = 0.15, SE = 0.07, 95% CI [0.01, 0.29], *t*_(215)_ = 1.98, *p* < 0.05), Table 3. However, within this grouping, all of the male subjects were collected on the day of their injury, while the female subjects were all collected the day after their injury occurred. In this instance, it seems more feasible that the interaction is a product of the female subjects being collected closer to the period of hyperreactivity found in Thibeault et al. (23), as opposed to a sex-related disparity.

**Table 3:** Mixed effect model results for BHI.

**Variable**	**β (SE)**	**95% CI**	***t*(DF) *p***
(Intercept)	0.41 (0.02)	[0.37, 0.44]	23.35(215) *p <* 0.001
Sex	−0.03 (0.03)	[−0.09, 0.04]	−0.73(215) *p >* 0.1
0–1 Days-Post	−0.02 (0.04)	[−0.09, 0.05]	−0.58(215) *p >* 0.1
2–3 Days-Post	**0.14 (0.03)**	**[0.08, 0.21]**	**4.44(224)** ***p <*** **0.001**
4–5 Days-Post	**0.09 (0.03)**	**[0.04, 0.15]**	**3.17(222)** ***p <*** **0.01**
6–7 Days-Post	**0.06 (0.03)**	**[0.01, 0.12]**	**2.32(231)** ***p <*** **0.05**
8–9 Days-Post	0.05 (0.03)	[0, 0.11]	1.81(231) *p >* 0.05
10–11 Days-Post	−0.01 (0.03)	[−0.07, 0.05]	−0.32(228) *p >* 0.1
12–13 Days-Post	0.01 (0.03)	[−0.05, 0.07]	0.24(223) *p >* 0.1
Sex:0–1 Days-Post	0.15 (0.07)	[0.01, 0.29]	1.98(215) *p <* 0.05
Sex:2–3 Days-Post	0.05 (0.07)	[−0.1, 0.12]	0.71(224) *p >* 0.1
Sex:4–5 Days-Post	0.05 (0.06)	[−0.05, 0.2]	0.82(222) *p >* 0.1
Sex:6–7 Days-Post	0 (0.06)	[−0.08, 0.17]	0.09(231) *p >* 0.1
Sex:8–9 Days-Post	−0.01 (0.06)	[−0.06, 0.16]	−0.23(231) *p >* 0.1
Sex:10–11 Days-Post	0.01 (0.06)	[−0.1, 0.12]	0.17(228) *p >* 0.1
Sex:12–13 Days-Post	0.08 (0.06)	[−0.05, 0.2]	1.18(223) *p >* 0.1

*The bold rows indicate significant effects or interactions*.

The overall longitudinal profile found in Thibeault et al. (23) was also predicted here by main effects for days-post 2–3 (β = 0.14, SE = 0.03, 95% CI [0.08, 0.21], *t*_(224)_ = 4.44, *p* < 0.001), 4–5 (β = 0.09, SE = 0.03, 95% CI [0.04, 0.15], *t*_(222)_ = 3.17, *p* < 0.01), and 6–7 (β = 0.06, SE = 0.03, 95% CI [0.01, 0.12], *t*_(231)_ = 2.32, *p* < 0.05), see Figure 1B.

#### PI

The model for PI had total explanatory power (conditional *R*^2^) of 72.02%, in which the fixed effects explain 8.35% of the variance (marginal R^2^). Here, a main effect of sex was predicted (β = −0.13, SE = 0.04, 95% CI [−0.21, −0.04], *t*_(195)_ = −2.96, *p* < 0.01). Exploring the population results in Figure 2C suggest that this effect may be a product of an inherent difference between males and females in the control population, as opposed to a sex-related difference. This is supported by the lack of significant interactions between sex and days-post injury grouping, Table 4. There was a significant main effect found at days-post 8–9 (β = 0.07, SE = 0.03, 95% CI [0.01, 0.13], *t*_(227)_ = 2.14, *p* < 0.05), that cannot be fully explained.

**Table 4:** Mixed effect model results for PI.

**Variable**	**β (SE)**	**95% CI**	**t(DF) p**
(Intercept)	0.77 (0.02)	[0.73, 0.81]	35.95(195) *p <* 0.001
Sex	**−0.13 (0.04)**	**[−0.21,−0.04]**	**−2.96(195)** ***p <*** **0.01**
0–1 Days-Post	−0.05 (0.04)	[−0.13, 0.02]	−1.33(186) *p >* 0.1
2–3 Days-Post	0.03 (0.04)	[−0.04, 0.09]	0.8(205) *p >* 0.1
4–5 Days-Post	0.02 (0.03)	[−0.04, 0.08]	0.62(211) *p >* 0.1
6–7 Days-Post	0.06 (0.03)	[0, 0.12]	1.99(228) *p* = 0.05
**8–9 Days-Post**	**0.07 (0.03)**	**[0.01, 0.13]**	**2.14(227)** ***p <*** **0.05**
10–11 Days-Post	0.03 (0.03)	[-0.04, 0.09]	0.8(220) *p >* 0.1
12–13 Days-Post	0.05 (0.04)	[-0.01, 0.12]	1.55(205) *p >* 0.1
Sex:0–1 Days-Post	0.05 (0.08)	[−0.1, 0.2]	0.68(186) *p >* 0.1
Sex:2–3 Days-Post	0.11 (0.07)	[−0.09, 0.16]	1.54(205) *p >* 0.1
Sex:4–5 Days-Post	0.05 (0.06)	[0, 0.27]	0.72(211) *p >* 0.1
Sex:6–7 Days-Post	0.08 (0.06)	[−0.02, 0.24]	1.23(228) *p >* 0.1
Sex:8–9 Days-Post	0.01 (0.07)	[−0.08, 0.17]	0.21(227) *p >* 0.1
Sex:10–11 Days-Post	0.04 (0.07)	[−0.09, 0.16]	0.56(220) *p >* 0.1
Sex:12–13 Days-Post	0.14 (0.07)	[0, 0.27]	1.96(205) *p >* 0.05

*The bold rows indicate significant effects or interactions*.

#### P2R

The model predicting P2R had a total explanatory power (conditional *R*^2^) of 74.42%, in which the fixed effects explain 10.32% of the variance (marginal *R*^2^). A main effect of sex was present (β = 0.05, SE = 0.02, 95% CI [0.01, 0.10], *t*_(196)_ = 2.21, *p* < 0.05). However, similar to PI, Figure 2D illustrates a difference between control groups. There were no significant interactions between sex and days-post found, see Table 5.

**Table 5:** Mixed effect model results for P2R.

**Variable**	**β (SE)**	**95% CI**	***t*(DF) *p***
(Intercept)	0.78 (0.01)	[0.75, 0.8]	62.97(196) *p <* 0.001
Sex	**0.05 (0.02)**	**[0.01, 0.1]**	**2.21(196)** ***p <*** **0.05**
0–1 Days-Post	0.04 (0.02)	[0, 0.08]	1.69(185) *p >* 0.05
2–3 Days-Post	0.01 (0.02)	[−0.02, 0.05]	0.72(205) *p >* 0.1
4–5 Days-Post	0 (0.02)	[−0.03, 0.04]	0.17(210) *p >* 0.1
6–7 Days-Post	0 (0.02)	[−0.03, 0.04]	0.28(227) *p >* 0.1
8–9 Days-Post	−0.01 (0.02)	[−0.05, 0.02]	−0.57(226) *p >* 0.1
10–11 Days-Post	0 (0.02)	[−0.03, 0.04]	0.16(220) *p >* 0.1
12–13 Days-Post	0 (0.02)	[−0.03, 0.04]	0.14(204) *p >* 0.1
Sex:0–1 Days-Post	0.02 (0.04)	[−0.06, 0.11]	0.42(185) *p >* 0.1
Sex:2–3 Days-Post	0.01 (0.04)	[−0.06, 0.09]	0.17(205) *p >* 0.1
Sex:4–5 Days-Post	0 (0.04)	[−0.1, 0.05]	0.09(210) *p >* 0.1
Sex:6–7 Days-Post	0 (0.04)	[-0.07, 0.08]	0.08(227) *p >* 0.1
Sex:8–9 Days-Post	0.01 (0.04)	[−0.07, 0.07]	0.31(226) *p >* 0.1
Sex:10–11 Days-Post	0.01 (0.04)	[−0.06, 0.09]	0.39(220) *p >* 0.1
Sex:12–13 Days-Post	−0.03 (0.04)	[−0.1, 0.05]	−0.65(204) *p >* 0.1

*The bold rows indicate significant effects or interactions*.

#### Correlations

**Figure 4:**
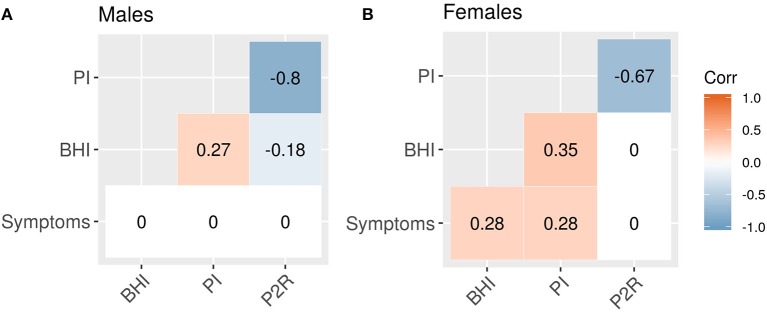
Feature correlations. **(A)** Male correlations. **(B)** Female correlations. Correlations that are not significant at the 0.05 level are set to zero.

The correlations between features provides additional information about the sex-related differences in this population, Figures 4A,B. Both sexes had significant negative correlations between PI and P2R (*r*_male_ = −0.8, *p* < 0.001; *r*_female_ = −0.67, *p* < 0.001). For the male population there were significant correlations between BHI and PI (*r* = 0.27, *p* < 0.001), as well as BHI and P2R (−0.18, *p* = 0.02), that were no present in the female population, Table 6. Conversely, the female population had significant correlations between symptoms and BHI (*r* = 0.28, *p* < 0.01), as well as symptoms and PI (*r* = 0.28, *p* < 0.01), that were not found in the male population, Table 6.

**Table 6:** Feature Correlations for the male and female groups.

	**BHI**	**PI**	**P2R**
**MALE GROUP**			
Symptoms	0.05 (*p =* 0.5)	−0.12 (*p =* 0.12)	0.14 (*p =* 0.07)
BHI		0.27 (*p <* 0.001)	−0.18 (*p <* 0.05)
PI			−0.8 (*p <* 0.001)
**FEMALE GROUP**			
Symptoms	0.28 (*p <* 0.01)	0.28 (*p <* 0.01)	−0.11 (*p <* 0.05)
BHI		0.35 (*p <* 0.01)	−0.01 (*p >* 0.05)
PI			−0.67 (*p <* 0.001)

Further exploring the sex-related correlation structure by an ANCOVA analysis reveals a significant difference in the slopes of the regression lines for symptoms and BHI between sexes (β = 58.55, SE = 19.07, 95% CI [20.98, 96.12], *t* = 3.07, *p* < 0.01). A difference in slopes was also found comparing symptoms and PI (β = 69.08, SE = 17.58, 95% CI [34.45, 103.70], *t* = 3.93, *p* < 0.001). For symptoms and P2R however, there was no significant difference in slope (β = −56.21, SE = 31.79, 95% CI [−118.82, 6.40], *t* = −1.77, *p* = 0.08), instead a difference in y-intercepts was found (β = 69.45, SE = 25.11, 95% CI [20.00, 118.91], *t* = 2.77, *p* < 0.01).

## Discussion

### Symptoms

Several studies have found a similar increase in self-reported symptoms for female subjects (1, 2). In the study from Baker et al. (4), the increased symptoms in the acute stage may have influenced recovery time—explaining the prolonged recovery for females. Although the difference between sexes here does appear more pronounced, comparing that difference to those other studies is not possible given the heterogeneity of the symptom collection.

The mechanism of injury presents a potentially confounding factor. In this study the majority of male subjects played helmeted sports (68%). The protection afforded by these helmets could have contributed to the overall lowered symptoms. However, in the study from Broshek et al. (1), female subjects were more than twice as likely to experience cognitive impairments than males in unhelmeted sports—illustrating that a difference existed even when accounting for helmets. The sex differences in reported symptoms observed in the current study were not accompanied by evidence for corresponding concussion-related differences in the TCD features. Moreover, the main effects of time observed for BHI suggest the progression of vascular injury to be similar for both sexes. A more compelling explanation would be an inherent reporting bias in the female group. Other studies have shown that female athletes tend to report more symptoms than males (7, 32). In addition, females are generally more focused on, and aware of, their health (33), suggesting that there is more of a motivation to ensure a complete recovery. Conversely, male athletes have a number of societal and cultural motivations to perceptually diminish the magnitude of their injury and return to sport as soon as possible (34). Similarly, it was shown in Kerr et al. (35), that male athletes were significantly more likely to hide a concussive injury.

There have been several studies exploring the physiological differences between sexes that contribute to the susceptibility and recovery from concussion, many of which center on the possible role of estrogen. In more severe traumatic brain injuries, estrogen has been shown to have a neuroprotective effect in male rats, but a deleterious one in females (36). In humans, the study from Gallagher et al. (5), found that female subjects suffering from a sport-related concussion who used hormonal contraceptives reported lower symptom severity than those who did not, suggesting that hormonal contraceptives may play a role in modulating the collapsing neurometabolic cascade that is a hallmark of concussive injuries (16). Another consistent theme in mTBI gender differences is decreased neck strength in women (37, 38), which has been shown to be inversely related to concussion susceptibility. A similar confound of this study is the role physical maturity plays in how someone responds to an mTBI. The study from Krix et al. (39) found that male subjects in early stages of puberty had increased odds of a prolonged recovery from a concussive injury. Although puberty clearly affects the adolescent brain (40), it is still unclear how that would contribute to the results of this work.

### TCD Features

It is important to note that in this context BHI is not meant an exact measure of reactivity. Breath-holding can introduce other autonomic and sympathetic responses that can confound its use for directly quantifying reactivity. However, as illustrated in Thibeault et al. (23), and confirmed by the main effects of days-post here, BHI as measured in this population, is a robust biomarker of mTBI. The difference in slopes of the regression lines between symptoms and BHI illustrate the vulnerability of relying on subjective measures alone.

The overall sex-related main effect for both PI and P2R is most surprising aspect of this analysis. For both features that effect did not appear to be based on the injury, but rather an inherent sex-related difference in this population. Previously, when sex was ignored both were altered immediately following an mTBI (23). The alterations of these features here, as illustrated in Figure 2, appear to only be present in the male population. PI is a complex metric that is influenced by the combinations of cerebral perfusion pressure, cerebrovascular resistance, arterial bed compliance, heart rate, and the pulse amplitude (26). Similarly, It has proposed that P2R is associated with distal bed compliance dynamics (41), however there is no established physiological correlation. Why either of these features would have a sex-related dependence is unclear and will need to be explored further in the future. Regardless, these results illustrate that that dependence is not due to the injury.

The study from Esposito et al. (42) showed that women had higher Cerebral Blood Flow (CBF) compared to males. Although, in the population here there was no significant trend in mean velocity during injury recovery, that may be because TCD cannot measure CBF directly, only the velocity. In addition, a post-concussive change in mean velocity has not been demonstrated with TCD (23).

## Conclusions

This is the first study to compare sex-related differences between clinical symptoms and TCD measurements in adolescent mTBI subjects. The objective measures highlight the need to mitigate patient heterogeneity when assessing concussion recovery and the discrepancy in clinical symptoms illustrates how difficult this can be for clinicians. In the case of males the possibility of under-reporting may need to be considered. A physiological measurement such as TCD may eventually help remove ambiguity and provide clinicians with an objective physiological measure of mTBI recovery.

## Data Availability

The raw data supporting the conclusions of this manuscript will be made available by the authors, without undue reservation, to any qualified researcher.

## Ethics Statement

This study was carried out in accordance with the recommendations of Western Institutional Review Board (IRB #20141111), with written informed consent from all subjects. All subjects gave written informed consent in accordance with the Declaration of Helsinki. The protocol was approved by the Western Institutional Review Board.

## Author Contributions

CT had full access to all the data in the study and takes responsibility for the integrity of the data and the accuracy of the data analysis. RH: study concept and design. CT and ST: analysis. CT, ST, and RH: interpretation of data. CT and ST: drafting of the manuscript. RH, SW, and ST: critical revision of the manuscript for important intellectual content. CT and ST: statistical analysis. NC and SW: technical or material support. RH and CT: study supervision.

### Conflict of Interest Statement

At the time that this research was conducted, CT, ST, NC, SW, and RH, were employees of, and either hold stock or stock options in, Neural Analytics, Inc.
